# Clinical and microbiologic characteristics of *tcdA*-negative variant *clostridium difficile* infections

**DOI:** 10.1186/1471-2334-12-109

**Published:** 2012-05-09

**Authors:** Jieun Kim, Hyunjoo Pai, Mi-ran Seo, Jung Oak Kang

**Affiliations:** 1 Department of Internal Medicine, Hanyang University College of Medicine, Guri, Korea; 2 Department of Internal Medicine, Hanyang University College of Medicine, Seoul, Korea; 3 Department of Laboratory Medicine, Hanyang University College of Medicine, Guri, Korea

**Keywords:** *Clostridium difficile* infection, *tcdA-*negative variant strain, Clinical outcome, Risk factor, Antimicrobial susceptibility test, *ermB* gene

## Abstract

**Background:**

The *tcdA-*negative variant (A^-^B^+^) of *Clostridium difficile* is prevalent in East Asian countries. However, the risk factors and clinical characteristics of A^-^B^+^*C. difficile* infections (CDI) are not clearly documented. The objective of this study was to investigate these characteristics.

**Methods:**

From September 2008 through January 2010, the clinical characteristics, medication history and treatment outcomes of CDI patients were recorded prospectively. Toxin characterization and antibiotic susceptibility tests were performed on stool isolates of *C. difficile*.

**Results:**

During the study period, we identified 22 cases of CDI caused by *tcdA-*negative *tcdB-*positive (A^-^B^+^) strains and 105 cases caused by *tcdA-*positive *tcdB-*positive (A^+^B^+^) strains. There was no significant difference in disease severity or clinical characteristics between the two groups. Previous use of clindamycin and young age were identified as significant risk factors for the acquisition of A^-^B^+^ CDI (OR = 4.738, 95% CI 1.48–15.157, *p* = 0.009 and OR = 0.966, 95% CI 0.935–0.998, *p* = 0.038, respectively) in logistic regression.

Rates of resistance to clindamycin were 100% and 69.6% in the A^-^B^+^ and A^+^B^+^ isolates, respectively (*p* = 0.006), and the *ermB* gene was identified in 17 of 21 A^-^B^+^ isolates (81%). Resistance to moxifloxacin was also more frequent in the A^-^B^+^ than in the A^+^B^+^ isolates (95.2% vs. 63.7%, *p* = 0.004).

**Conclusions:**

The clinical course of A^-^B^+^ CDI is not different from that of A^+^B^+^ CDI. Clindamycin use is a significant risk factor for the acquisition of *tcdA-*negative variant strains.

## Background

*Clostridium difficile* infection (CDI) is a major cause of healthcare-associated infections worldwide. While the hypervirulent ribotype 027 strain with binary toxin has led to outbreaks only in North America and Europe [1,2], toxin A-negative (A^-^B^+^) *C. difficile* infections have been reported worldwide [3-5]. The A^-^B^+^ strain infections occur in an epidemic or sporadic form worldwide, while A^-^B^+^ CDI is more frequent in East Asian countries [4,6-9].

Initially toxin A was considered to be the most important factor responsible for diarrheal disease [5]; however, several reports have documented that A^-^B^+^ strains can lead to similar symptoms, from mild diarrhea to severe pseudomembranous colitis [10,11]. The risk factors and clinical characteristics of A^-^B^+^ CDI have not been clearly documented.

In this study, we investigated the presence of A^-^B^+^ strains in healthcare-associated CDI (HA-CDI) in Korea, compared the clinical characteristics of A^-^B^+^ CDI and A^+^B^+^ (*tcdA*-positive *tcdB*-positive) CDI, and analyzed the risk factors for acquisition of A^-^B^+^ CDI. We also assessed the susceptibilities to clindamycin and moxifloxacin of the A^-^B^+^ and A^+^B^+^ strains.

## Methods

### Setting and study design

This study was conducted at Hanyang University Hospital, a 900-bed tertiary care facility located in Seoul, South Korea. From September 2008 through January 2010, we enrolled suspected HA-CDI patients and recorded prospectively their clinical characteristics. After isolation of *C. difficile* from patients’ stools, microbiologic studies were performed. To compare the effects of 'toxin A and toxin B' strains and 'toxin B alone' strains on clinical characteristics, patients infected with A^+^B^+^ and A^-^B^+^ strains were enrolled, while those with binary toxin-positive isolates were excluded. This study was approved by the institutional review board of Hanyang University Hospital (HYUH IRB 2010-R-12).

### Definitions

Diarrhea was defined as unformed stools more than 3 times per day on consecutive days, or 6 times within 36 h. CDI was diagnosed when patients had diarrhea and the *C. difficile* stool isolates contained toxin genes detected by multiplex PCR [12]. We diagnosed HA-CDI when CDI developed at least 72 h after hospitalization, or within 2 months of the last discharge if the patient did spend time in a healthcare facility [13].

Clinical cure was defined as resolution of diarrhea within the treatment period, and clinical failure as the need for treatment change to resolve the diarrhea. Resolution of diarrhea required conversion to two or fewer semi-formed or formed stools per day [14]. Recurrence was defined as growth of toxinogenic *C. difficile*, a positive A&B toxin assay (VIDAS® *C. difficile* Toxins A & B; BioMerieux SA, Marcy l’Etoile, France), or a pseudomembrane on endoscopy, with resurgence of symptoms after cessation of treatment at least 10 days after the first episode [15]. Global cure was defined as clinical cure without recurrence.

### Collection of data

The demographic and clinical data collected in the study were age, sex, length of hospital stay, medication history within the past 2 months, and underlying diseases including Charlson scores. Installation of a catheter, the use and dosage of antibiotics, probiotics, proton pump inhibitors, H2 blockers, steroids, chemotherapy, and surgical procedure within the past 2 months, were investigated as potential risk factors for CDI [16]. Past medication history was obtained from medical records. The amount of antibiotics administered was expressed as the number of defined daily doses (DDD), which means the total amount of antibiotic divided by the DDD [17].

Vital signs, stool character, stool frequency, and abdominal pain and tenderness were monitored within 24 h after inclusion. Laboratory tests such as white blood cell (WBC) count, albumin and C-reactive protein (CRP) levels were performed within 24 h of enrollment. Age >60 years, temperature >38.3°C, albumin level <2.5 mg/dL, and WBC count >15,000 cells/mm^3^ received 1 point each, and the scores were summed for each patient. Scores above 2 were defined as severe CDI [18,19].

### Microbiologic studies

Stool specimens were grown anaerobically on *C. difficile*-selective cycloserine-cefoxitin-taurocholate agar (CCFA-TA, Oxoid Ltd., Cambridge, UK), supplemented with 7% horse blood, after alcohol shock treatment [20]. Colonies of *C. difficile* were identified with API® Rapid ID 32A (BioMerieux SA, Marcy l’Etoile, France). ATCC 43598 and PCR-ribotype 027 (BI/NAP1/027) were used as internal controls.

Template DNA from stool isolates was used in multiplex PCR to investigate the presence of toxin genes, as described elsewhere, with minor modifications [12]. After the strains producing binary toxin were excluded by multiplex PCR, toxin typing was performed [21].

Minimum inhibitory concentrations (MICs) of clindamycin and moxifloxacin were measured on supplemented Brucella agar by the E-test (AB-BIOdisc, Solna, Sweden), as recommended by the Clinical and Laboratory Standards Institute (CLSI) [22]. *C. difficile* ATCC 700057 was used as quality control strain for susceptibility testing.

PCR was performed on template DNA to detect the *ermB* gene [23]. A PCR product of 688 base pairs on electrophoresis was considered a positive result.

### Statistical methods

SPSS version 18.0 for Windows (SPSS Inc., Chicago, IL, USA) was used for statistical analysis. Categorical variables were analysed using Pearson’s chi-square test or Fisher’s exact test. Continuous variables were analysed using an independent *t*-test or the Mann–Whitney *U*-test. Variables with *p*-values <0.1 on univariate analysis were included in the multivariate analysis. A *p*-value <0.05 in a two-tailed test of significance was considered statistically significant.

## Results

During the study period, 138 *C. difficile* isolates were obtained from HA-CDI patients; 11 isolates (8.0%) were confirmed by multiplex PCR to have binary toxin genes, 22 (15.9%) were A^-^B^+^ strains, and 105 (76.1%) were A^+^B^+^ strains. Consequently, 22 patients with A^-^B^+^ strains and 105 with A^+^B^+^ strains were enrolled.

### Comparison of the demographic and clinical characteristics of the A^-^B^+^ and A^+^B^+^ CDI groups

Demographic and clinical characteristics were compared in the 22 A^-^B^+^ patients and the 105 A^+^B^+^ patients (Table 1). There were no differences in age, sex, length of hospital stay, history of recent surgery or Charlson score between the two groups; however, more cases of chronic obstructive pulmonary disease (COPD) as an underlying disease were identified in the A^-^B^+^ group (*p =* 0.049). No differences were found between the two groups with regard to factors associated with the CDI severity score. Using a definition of severe CDI adapted from Zar et al. [18,19], severe CDI occurred at a similar rate in the A^+^B^+^ and the A^-^B^+^ group (19% and 18.2% respectively, *p* = 0.925). WBC counts and CRP levels were higher and the albumin level lower in the A^+^B^+^ CDI group, but these differences was not statistically significant. Clinical characteristics associated with CDI, such as stool frequency, mucoid stool, abdominal pain or tenderness, were not significantly different between the groups. The A^-^B^+^ group was 1.067 times more likely to develop pseudomembranous colitis than the A^+^B^+^ group, as measured by odds ratio.

**Table 1:** Demographic and clinical characteristics of patients infected with *Clostridium difficile tcdA*-positive *tcdB*-positive (A^+^B^+^) strains and with *tcdA-*negative *tcdB-*positive (A^-^B^+^) strains

		**A^+^B^+^ (n = 105)**	**A^-^B^+^ (n = 22)**	***p* value ^a^**
Sex (female)	N (%)	54 (51.4)	8 (36.4)	0.244
Age	Mean (SD)	65.2 (15.10)	58.5 (19.12)	0.143
Length of hospital stay	Mean (SD)	34.8 (44.41)	24.3 (21.20)	0.583
Charlson score	Mean (SD)	3.17 (2.482)	2.50 (2.345)	0.172
Underlying disease				
COPD	N (%)	8 (7.6)	5 (22.7)	0.049
CVA	N (%)	28 (26.7)	2 (9.1)	0.100
Surgical procedure	N (%)	28 (26.7)	2 (9.1)	0.100
Severity score	Mean (SD)	0.96 (0.746)	1.00 (0.873)	0.961
Old age ^b^	N (%)	67 (63.8)	12 (54.5)	0.472
Fever ^c^	N (%)	10 (9.5)	5 (22.7)	0.137
Hypoalbuminemia ^d^	N (%)	10 (9.5)	1 (4.5)	0.688
Leukocytosis ^e^	N (%)	14 (13.3)	4 (18.2)	0.515
Severe CDI ^f^	N (%)	20 (19.0)	4 (18.2)	0.925
Clinical findings				
Pain	N (%)	25 (23.8)	10 (45.5)	0.063
Tenderness	N (%)	36 (34.3)	10 (45.5)	0.338
Stool Fr ≥10/day	N (%)	21 (20.4)	2 (9.1)	0.362
Mucoid stool	N (%)	19 (18.1)	8 (36.4)	0.083
Laboratory findings				
WBC (cells/mm^3^)	Mean (SD)	10892 (5527)	10263 (7746)	0.239
Albumin (mg/dL)	Mean (SD)	3.1 (0.57)	3.4 (0.53)	0.073
CRP (mg/dL)	Mean (SD)	7.40 (6.397)	5.98 (6.194)	0.139
Pseudomembrane ^g^	N (%)	10/26 (38.5)	2/5 (40.0)	

A^+^B^+^, *tcdA*-positive *tcdB*-positive strain; A^-^B^+^, *tcdA-*negative *tcdB-*positive strain; COPD, chronic obstructive pulmonary disease; CVA, cerebrovascular accident; ICU, intensive care unit; Fr, frequency; CDI, *Clostridium difficile* infection; WBC, white blood cell; CRP, C-reactive protein.

^a^ From Fisher’s exact test for categorical variables or the Mann–Whitney *U*-test for continuous variables.

^b^ Defined as age >60 years.

^c^ Defined as temperature >38.3°C.

^d^ Defined as albumin level <2.5 mg/dL.

^e^ Defined as a WBC count >15,000 cells/mm.^3^

^f^ Severity scores >2 points were regarded as severe CDI.

^g^ Denominator comprises patients examined by endoscopy.

### Comparison of outcomes in the A^-^B^+^ and A^+^B^+^ CDI groups

Of the 127 patients with HA-CDI, 7 were discharged before diagnosis and 3 died from an underlying disease before they had begun treatment. In 32 patients, diarrhea improved or resolved without treatment, and 85 patients (16 A^-^B^+^ and 69 A^+^B^+^ CDI cases) completed treatment (16/22 vs. 69/105, *p* = 0.623). Treatment outcomes were compared in the two groups. The treatment regimens (metronidazole or vancomycin) did not differ significantly (*p* = 0.681). The rates of global cure, failure and mortality were respectively 62.3%, 5.8% and 10.1% in the A^+^B^+^ group and 75%, 12.5% and 12.5% in the A^-^B^+^ group (*p* = 0.398, 0.315, and 0.675, respectively) (Table 2). One case of mortality attributable to CDI occurred in the A^+^B^+^ group. Additionally 15 of 69 patients (21.7%) in the A^+^B^+^ group and none in the A^-^B^+^ group (0/16) experienced CDI recurrence after completion of treatment; however, the difference in recurrence rate between the groups did not reach statistical significance (*p* = 0.063). Clinical outcomes were not significantly different between the metronidazole and vancomycin treatment groups (measured in all patients, *p* for trend = 0.597).

**Table 2:** Comparison of clinical outcomes in patients infected with *tcdA*-positive *tcdB*-positive (A^+^B^+^) strains of *Clostridium difficile* and those infected with *tcdA-*negative *tcdB-*positive (A^-^B^+^) strains

**Clinical outcome**	**Group**		***p* value**
	**A^+^B^+^ (n (%))**	**A^-^B^+^ (n (%))**	
Global cure	43 (62.3)	12 (75.0)	0.398
Failure	4 (5.8)	2 (12.5)	0.315
Death	7 (10.1)	2 (12.5)	0.675
Recurrence	15 (21.7)	0 (0.0)	0.063
Total	69 (100.0)	16 (100.0)	

A^+^B^+^, *tcdA*-positive *tcdB*-positive strain; A^-^B^+^, *tcdA*-negative *tcdB*-positive strain.

### Comparison of previous medication history in the A^-^B^+^ and A^+^B^+^ groups

Medication histories of the patients were obtained by retrospective review of their medical records. After excluding 5 cases of HA-CDI due to incomplete medical records, 21 cases of A^-^B^+^ CDI and 101 cases of A^+^B^+^ CDI were included in the analysis (Table 3).

**Table 3:** Previous medication in patients infected with *tcdA*-positive *tcdB*-positive (A^+^B^+^) strains of *Clostridium difficile* and with *tcdA-*negative *tcdB-*positive (A^-^B^+^) strains

		**A^+^B^+^ (n = 101)**	**A^-^B^+^ (n = 21)**	***P* value ^a^**
Medication				
PPI	N (%)	35 (34.7)	6 (28.6)	0.800
H2 blockers	N (%)	49 (48.5)	8 (38.1)	0.474
Probiotics	N (%)	49 (48.5)	13 (61.9)	0.339
Steroids	N (%)	37 (36.6)	7 (33.3)	1.000
Chemotherapy	N (%)	12 (11.9)	3 (14.3)	0.722
Antibiotics				
All-DDD	Mean (SD)	25.4 (18.91)	27.0 (21.83)	0.989
Days of antibiotic use	Mean (SD)	18.7 (12.25)	16.6 (12.23)	0.317
Antibiotics use				
Fluoroquinolones	N (%)	46 (45.5)	14 (66.7)	0.096
2nd Quinolones	N (%)	22 (21.8)	8 (38.1)	0.161
3rd Quinolones	N (%)	34 (33.7)	9 (42.9)	0.458
Clindamycin	N (%)	17 (16.8)	11 (52.4)	0.001
ESC	N (%)	57 (56.4)	11 (52.4)	0.811
BL/BLI	N (%)	36 (35.6)	6 (28.6)	0.620
Carbapenem	N (%)	10 (9.9)	2 (9.5)	1.000
Glycopeptides	N (%)	20 (19.8)	2 (9.5)	0.360
Metronidazole	N (%)	29 (28.7)	3 (14.3)	0.275
Antibiotics dose				
Fluoroquinolones	Mean (SD)	5.606 (9.2145)	7.138 (10.0394)	0.202
2nd Quinolones	Mean (SD)	1.858 (5.1504)	2.210 (4.7322)	0.168
3rd Quinolones	Mean (SD)	3.737 (7.2372)	4.929 (9.3117)	0.484
Clindamycin	Mean (SD)	1.111 (3.1081)	4.302 (5.1853)	<0.0001
ESC	Mean (SD)	5.260 (7.7728)	3.595 (4.5188)	0.611
BL/BLI	Mean (SD)	3.825 (9.2440)	3.536 (8.2533)	0.611
Carbapenem	Mean (SD)	0.636 (3.3368)	0.524 (1.7210)	0.995
Glycopeptides	Mean (SD)	1.255 (3.4253)	0.548 (1.9359)	0.270
Metronidazole	Mean (SD)	2.850 (6.9772)	1.397 (3.7901)	0.194

A^+^B^+^, *tcdA*-positive *tcdB*-positive strain; A^-^B^+^, *tcdA-*negative *tcdB-*positive strain; PPI, proton pump inhibitor; DDD, defined daily dose; ESC, extended-spectrum cephalosporins; BL/BLI, beta-lactam/beta-lactamase inhibitor.

^a^ From Fisher’s exact test for categorical variables or the Mann–Whitney *U*-test for continuous variables.

Previous use of proton pump inhibitors, H2 blockers, probiotics, steroids or chemotherapy was not significantly different between the groups. The most commonly prescribed antibiotics in the A^+^B^+^ CDI group were extended-spectrum cephalosporins (ESCs) (56.4%) followed by fluoroquinolones (45.5%) and beta-lactam/beta-lactamase inhibitors (35.6%), while in the A^-^B^+^ group they were fluoroquinolones (66.7%), clindamycin (52.4%) or ESCs (52.4%). Calculating the amount of antibiotics as DDD showed that, in the A^+^B^+^ group, the order of antibiotics by quantity was similar to the order by frequency of use. In the A^-^B^+^ group, antibiotics consumed in largest quantities were fluoroquinolones, clindamycin and ESCs. Finally, a comparison of previous use and amount of antibiotics in the two groups showed that both the use and amount of clindamycin were significantly higher in the A^-^B^+^ group (*p* = 0.001 and *p* < 0.0001, respectively).

### Risk factors for A^-^B^+^ strain acquisition

We performed a multivariate logistic regression analysis to identify the risk factors for acquisition of *tcdA*-negative strains. Age, sex, chronic obstructive pulmonary disease as an underlying disease, albumin level, and quinolone and clindamycin use were included in the analysis. Previous use of clindamycin was found to be a significant risk factor for A^-^B^+^*C. difficile* infection (OR = 4.738, 95% CI 1.481–15.157, *p* = 0.009). Age also had a statistically significant effect (OR = 0.966, 95% CI 0.935–0.998, *p* = 0.038) (Table 4).

**Table 4:** Multivariate analysis of risk factors for *tcdA-*negative *tcdB-*positive (A^-^B^+^) *Clostridium difficile* infections

	**OR**	**95% CI for Exp(B)**		***p* value**
		**lower**	**upper**	
Age	0.966	0.935	0.998	0.038
Sex	1.436	0.482	4.273	0.516
Albumin	2.584	0.901	7.412	0.077
COPD	2.269	0.462	11.153	0.313
Fluoroquinolone use	1.808	0.529	6.180	0.345
Clindamycin use	4.738	1.481	15.157	0.009

OR, odds ratio; CI, confidence interval; COPD, chronic obstructive pulmonary disease.

### Microbiologic studies

Toxinotyping was performed on the 138 isolates of C. difficile; 104 of the 105 A^+^B^+^ isolates (99.0%) belonged to toxinotype 0, and one isolate to toxinotype I; all 22 isolates of the A^-^B^+^ strain were toxinotype VIII; 8 of 11 binary toxin-producing isolates were toxinotype IV and 3 isolates, toxinotype III.

Clindamycin and moxifloxacin susceptibility testing was performed on only 123 isolates (21 A^-^B^+^ isolates and 102 A^+^B^+^ isolates) because subculture failed in the case of 4 isolates.

The A^-^B^+^ strains had significantly higher resistance rates to clindamycin and moxifloxacin than the A^+^B^+^ strains (p = 0.006 and 0.004, respectively). In the A^-^B^+^ group, the resistance rates to clindamycin and moxifloxacin were 100% (MIC range 16 6– > 256 mg/L) and 95.2% (MIC range 2–32 mg/L), respectively, whereas in the A^+^B^+^ group they were 69.6% and 63.7%, respectively, with wide ranges of MIC (MIC range 1.5– > 256 mg/L and <0.25–128 mg/L, respectively). Resistance to moxifloxacin was correlated with resistance to clindamycin (P < 0.0001). The moxifloxacin resistance rate among clindamycin-resistant A^+^B^+^ isolates was 88.7% (63/71) compared with 6.5% (2/31) among clindamycin-susceptible A^+^B^+^ isolates. By the same token, the clindamycin resistance rate for the moxifloxacin-resistant A^+^B + isolates was 96.9% (63/65), but only 21.6% (8/37) for the moxifloxacin-susceptible A^+^B^+^ isolates.

*ErmB* gene-specific PCR revealed that 17 of the 21 A^-^B^+^ isolates (81.0%) were *ermB*-positive. All 17 of these isolates were resistant to clindamycin, and 16 (94.1%) showed high level resistance (MIC > 256 mg/L). Among the 102 A^+^B^+^ isolates, there were 49 (48.0%) *ermB*-positive isolates, 37 of which (75.5%) were highly resistant to clindamycin.

## Discussion

The prevalence of toxin A-negative/toxin B-positive (A^-^B^+^) strains among CDI isolates varies depending on the country. In most of Europe and in North America, the prevalence of A^-^B^+^ strains has been reported to be only 0.2–8% [6,24]; however, such strains are thought to have caused several outbreaks in those regions. For example, in Poland the prevalence of A^-^B^+^ strains has increased from 11% to 45% [25] since there was an outbreak. By contrast, Japan has reported a high prevalence of A^-^B^+^ strains; the peak incidence was 39% in 2000 [10], and the incidence decreased to 12.7% in 2005–2008 [8]. A report from Shanghai revealed that A^-^B^+^ strains were responsible for a third of all CDI cases [4]. In Korea, the frequency of A^-^B^+^ strains was reported to be 4.9% in 1998 [26], and it then increased to 50.9% in 2004–2005 [7,11]. In our study, the frequency of A^-^B^+^ strains among the HA-CDI patients in 2009 was 15.9%. Even given the expected fluctuations in time and space, such a high frequency of A^-^B^+^ strains over the East Asian region is interesting. Possible explanations could be similar profiles of antibiotic use, and spread of the pathogen through common foods, as well as the heavy traffic of people across the three countries.

The role of toxin A and toxin B in the pathogenecity of CDI has been debated. Although early studies suggested that toxigenic strains of *C. difficile* always produce both toxin A and toxin B [5], one study showed that toxin B is the key virulence factor [27]. However, a recent study figured out that *C. difficile* producing either one or both toxins showed cytotoxic activity in vitro that translated directly into virulence in vivo [28]. Several studies of the clinical characteristics of A^-^B^+^ CDI [8,29-31] have shown that A^-^B^+^ strains cause a wide spectrum of diseases from asymptomatic colonization to life-threatening colitis [31], and that there is no significant difference between the clinical manifestations and outcomes of CDI caused by A^-^B^+^ and A^+^B^+^ strains [10]. However, another study reported more cases of pseudomembranous colitis on endoscopy in A^-^B^+^ patients than in A^+^B^+^ patients (70% vs. 40%, *p* = 0.0016) [11]. Furthermore, severe CDI caused by PCR ribotype 017 was responsible for 5% of cases in Germany, and all these cases had lethal outcomes [30]. In another report, the mortality during a PCR ribotype 017 CDI outbreak attributable to that ribotype was similar to that attributable to PCR ribotype 027 (7.0% vs. 6.5%), and higher than that attributable to other types (7.0% vs. 1.6%) [32]. Conversely, in our study there was no significant difference in the clinical characteristics, the laboratory findings or the incidence of pseudomembranous colitis between the study groups, and there were no deaths attributable to A^-^B^+^ CDI. The clinical outcomes also did not differ in the two groups, although there was no A^-^B^+^ CDI recurrence. In previous reports, the recurrence rate of A^-^B^+^ CDI varied from 9 to 35.7% and was similar to that of A^+^B^+^ CDI [3,29]. The reason that we observed no recurrence and no attributable death could be simply that the number of A^-^B^+^ cases in this non-epidemic setting was too low. Alternatively it could be because the A^-^B^+^ CDI developed in younger patients with lower Charlson scores, or because the shorter hospital stays among the A^-^B^+^ group prevented re-infection (however that effect was not statistically significant). Another possibility is that A^-^B^+^ CDI recurs less frequently than A^+^B^+^ CDI. To investigate this, a further study with a larger number of cases would be necessary.

In order to confirm that clindamycin use is an important risk factor for A^-^B^+^ CDI in a clinical setting, we compared the antimicrobial susceptibilities of the A^-^B^+^ and A^+^B^+^ strains. As expected [33], all 21 A^-^B^+^ strains were resistant to clindamycin. High level resistance to clindamycin (MIC > 256 mg/L) in *C. difficile* is usually linked to the *ermB* gene, encoding resistance to macrolide-lincosamide-streptogramin B (MLS_B_) [31]. In this study, the percentage of *ermB*-positive strains was 53.7% among all *C. difficile* isolates and 81.0% among the A^-^B^+^ isolates. Among the A^-^B^+^ strains, 4 of the 21 isolates were *ermB-*negative, and all of these were highly resistant to clindamycin. Clindamycin resistance in these strains could also be induced by other mechanisms such as other *erm* genes, efflux mechanisms, or nucleotide substitutions in other genes [34]. Further study of these resistance mechanisms would be desirable.

In previous studies, moxifloxacin resistance was closely associated with clindamycin resistance (95% CI 68–97%) [34,35]. It was reported that, of isolates resistant to fluoroquinolones, 98% were resistant to either erythromycin or clindamycin, although fluoroquinolone resistance in the absence of MLS_B_ resistance was rare [35]. Similarly, in our study, resistance to moxifloxacin was highly correlated with clindamycin resistance (*p* < 0.0001). An efflux pump may be responsible for the co-resistance to the MLS_B_ drugs and fluoroquinolone.

## Conclusions

The prevalence of A^-^B^+^ strains among HA-CDI patients in Korea was 15.9%. Disease severity and clinical characteristics were not significantly different between the A^-^B^+^ and the A^+^B^+^ patients. Previous use of clindamycin is a risk factor for acquisition of an A^-^B^+^ strain, and all tested A^-^B^+^ isolates were highly resistant to clindamycin. Heavy use of clindamycin appears to facilitate A^-^B^+^ strain infection in our hospitals.

## Competing interests

The authors declare that they have no competing interests.

## Authors’ contributions

J Kim contributed to protocol preparation, data collection and analysis, as well as manuscript preparation. H Pai provided the funding for this study and made a major contribution to the interpretation of data and appraisal of the manuscript. M Seo participated in the microbiologic studies, and J O Kang contributed to the microbiologic studies, discussion of results and revision of the manuscript. All authors read and approved the final manuscript.

## Pre-publication history

The pre-publication history for this paper can be accessed here:

http://www.biomedcentral.com/1471-2334/12/109/prepub
